# CRISPR/Cas9 Delivery System Engineering for Genome Editing in Therapeutic Applications

**DOI:** 10.3390/pharmaceutics13101649

**Published:** 2021-10-09

**Authors:** Hao Cheng, Feng Zhang, Yang Ding

**Affiliations:** 1 Key Laboratory of Drug Quality Control and Pharmacovigilance, China Pharmaceutical University, Ministry of Education, Nanjing 210009, China; chenghaopharm@stu.cpu.edu.cn (H.C.); 3220010156@stu.cpu.edu.cn (F.Z.); 2 State Key Laboratory of Natural Medicines, Department of Pharmaceutics, China Pharmaceutical University, Nanjing 210009, China; 3 NMPA Key Laboratory for Research and Evaluation of Pharmaceutical Preparations and Excipients, China Pharmaceutical University, Nanjing 210009, China

**Keywords:** CRISPR/Cas9, genome editing, site-specific trafficking, overcome off-target risks, therapeutic applications

## Abstract

The clustered regularly interspaced short palindromic repeats (CRISPR)/associated protein 9 (CRISPR/Cas9) systems have emerged as a robust and versatile genome editing platform for gene correction, transcriptional regulation, disease modeling, and nucleic acids imaging. However, the insufficient transfection and off-target risks have seriously hampered the potential biomedical applications of CRISPR/Cas9 technology. Herein, we review the recent progress towards CRISPR/Cas9 system delivery based on viral and non-viral vectors. We summarize the CRISPR/Cas9-inspired clinical trials and analyze the CRISPR/Cas9 delivery technology applied in the trials. The rational-designed non-viral vectors for delivering three typical forms of CRISPR/Cas9 system, including plasmid DNA (pDNA), mRNA, and ribonucleoprotein (RNP, Cas9 protein complexed with gRNA) were highlighted in this review. The vector-derived strategies to tackle the off-target concerns were further discussed. Moreover, we consider the challenges and prospects to realize the clinical potential of CRISPR/Cas9-based genome editing.

## 1. Introduction

The clustered regularly interspaced short palindromic repeats (CRISPR)/associated protein 9 (CRISPR/Cas9) as a revolutionary genome editing technology has achieved rapid development and great attention in recent years for the splendid promise in genetic disorder treatment. The native CRISPR/Cas system was served as an adaptive immune system in archaea and bacteria to protect them from invasive nucleic acids. The CRISPR was originally discovered as a set of repeated short sequences in the genome of Escherichia coli (*E. coli*) in 1987 [1]. However, this accidental discovery did not attract enough attention until the CRISPR-associated protein (Cas) was identified. The Cas gene was located adjacent to the CRISPR gene, indicating their functional associations. In 2005, Mojica et al. firstly proposed the hypothesis that CRISPR and Cas protein are related to the adaptive immune defense system [2]. They speculated that antisense RNAs were employed in this system as memory features of past invasions. In 2012, Doudna and Charpentier figured out that CRISPR RNA (crRNA) was base-paired to transactivating-crRNA (tracrRNA) to form a two-RNA structure named guide RNA (gRNA) and directed the Cas9 nuclease to the target site [3]. In 2013, Zhang et al. and Church et al. demonstrated gene editing in mammalian cells with CRISPR/Cas9, providing a programmable tool for genomic surgery [4]. Particularly, the 2020 Nobel Prize in chemistry was awarded to Doudna and Charpentier for their fundamental studies of CRISPR/Cas9 technology.

**Figure 1:**
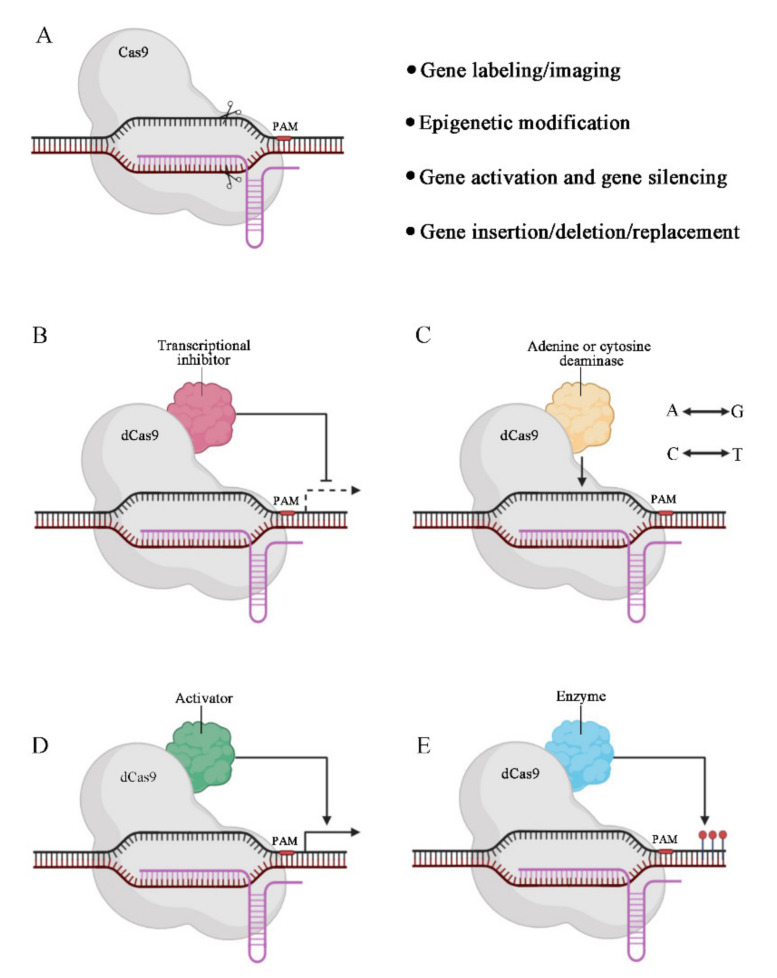
Applications of CRISPR/Cas9 system. (**A**) Schematic illustration of the molecular mechanism of CRISPR/Cas9 system. (**B**) The dCas9 fused to a transcriptional inhibitor (red shape) can repress transcription. (**C**) A DNA base editor consists of a dCas9 and an adenine or cytosine deaminase (yellow) that converts A to G or T to C, respectively. (**D**) The dCas9 fused to transcriptional activator (green) can boost transcription. (**E**) The dCas9 fused with enzymes (blue) that can modify epigenetic marks of DNA can be used to change gene expression status (Created with BioRender.com, accessed on 5 September 2021).

The CRISPR/Cas system possesses two crucial components, including gRNA and Cas protein, to enable effective genome editing in eukaryotic cells. Currently, Cas9 derived from *Streptococcus pyogenes* Cas9 (SpCas9) is the most concerned endonuclease variant due to high target specificity and simplicity [5]. The Cas9 shares two active sites termed HNH and RuvC, which could cleavage double-stranded DNA (dsDNA) and create site-specific double-strand breaks (DSB) [6] (Figure 1A). The HNH nuclease site cuts the DNA strand complementary to crRNA, whereas the RuvC domain cleavages the opposite. The binding site of Cas9 is located at the upstream of the protospacer-adjacent motif (PAM) containing the 5′-NGG base sequence. Moreover, the separated crRNA and tracrRNA could be combined into a simple single-guide RNA (sgRNA) [3]. Upon the guidance of sgRNA, Cas9 protein can specifically target any genomic locus via base pairing and induce a DSB. Then, cells exploit either non-homologous end-joining (NHEJ) or homology-directed repair (HDR) mechanism to repair the damaged genome [7]. Generally, the NHEJ pathway contributes to insertions or deletions (indels) at the site of cleavage, while HDR could induce defined alterations with a DNA donor.

Prior to the CRISPR/Cas9 system, DNA recognition domain-containing endonucleases including meganucleases, zinc-finger nucleases (ZFNs), and transcription activator-like effectors (TALENS) were the dominating tools for gene editing [8]. The earliest meganucleases possess varying DNA sequence specificity, which was enabled by protein engineering of recognition sites. The complexity of engineering endonucleases and low cutting efficiency hindered the promotion. Subsequent ZFNs and TALENs further unlock the potential of genome engineering. They simultaneously consisted of a non-specific FokI nuclease domain and tailor-made DNA recognition domains. Nevertheless, ZFNs and TALENs should bind to the targeted DNA with appropriate spacing and correct orientation for the dimerization of FokI endonucleases. Since the methods employed engineered proteins to recognize targeted DNA, a specific protein was necessary to be designed for the targeting, which is labor and time intensive. Alternatively, CRISPR/Cas9 has emerged as a preferable technology for gene manipulation due to obviating the redesign of protein components. The technology can adapt to different genome sites via simply the design of around 20 nucleotide target binding parts of sgRNA. Moreover, the CRISPR/Cas9 system is insensitive to base methylation in target sites and allows cleaving multiple sites by co-delivering diverse sgRNA [9].

The CRISPR/Cas9 system shares tremendous promise for various applications including gene correction, transcriptional regulation, disease modeling, and nucleic acids imaging. For instance, the CRISPR/Cas9 technology has been exploited to correct oncogenic mutations, and reverse the monogenic disorders permanently. CRISPR/Cas9 was further confirmed as a therapeutic potential in polygenic diseases such as a viral infection. Moreover, researchers have successfully used this technology to establish disease models [10]. Establishing disease models was an expensive and laborious process in the past, since it required tedious embryonic stem cell manipulation and endless mouse husbandry to obtain the desirable phenotype and genotype. In addition, the Cas9 protein has been re-engineered to extend the applications of CRISPR/Cas9 system (Figure 1B–E). Especially, the Cas9 protein with mutations in two nuclease domains (dead Cas9, dCas9) could maintain the CRISPR/Cas9 system with potential of epigenetic regulation. CRISPR interference (CRISPRi) involving dCas9 fused with repressors can mediate gene silence, while CRISPR activation (CRISPRa) takes advantage of dCas9 and transcription activators to realize transcriptional activation [11]. The dCas9 derived gene editors can change base types and are suitable for correcting point mutations [12,13]. In addition, dCas9 fused with fluorescent proteins such as red fluorescent protein (RFP) and green fluorescent protein (GFP) is capable of visualizing nucleic acids and realizing live-cell imaging [14].

Owing to the merits of accuracy and simplicity, CRISPR-Cas9 has revolutionized the field of gene therapy, and displayed inspiring effects for multiple usages. However, the insufficiency of rational delivery strategies is still a hurdle in clinical translation. Traditional CRISPR-Cas9 physical delivery methods include microinjection and electroporation [15,16], which perform high transfection efficacy and stable uptake mechanism. However, the introduction of microinjection and electroporation technologies are harmful to the cell membrane due to individual cell-manipulation. Direct physical methods are effective and promising to edit specific cells, yet most of the tissues are not suitable for ex vivo transfection. This review highlights rationally designed viral and non-viral vectors, and provides prospects for future CRISPR-Cas9 research.

## 2. Clinical CRISPR/Cas9 Delivery Strategies: Physical Import and Viral Vector Transfection

### 2.1. Clinical Applications of CRISPR-Based Genome Editing

The CRISPR/Cas9 system has emerged as a powerful tool to manipulate the genome for therapeutic purposes. After screening out clinical trials involving the CRISPR/Cas9 technology for gene identification, gene function exploration, and disease model establishment, the critical information of 25 clinical trials including NTC identifier, study phase, and targeted conditions, has been summarized in Table 1. The current clinical investigations mainly focus on typical hereditary diseases (e.g., hemophilia, thalassemia, sickle cell anemia), cancer immunotherapy, and viral infection inhibition. The CRISPR/Cas9-based clinical trials could be classified into two main clusters: Adoptive cell therapy (ACT) and in vivo therapy. In ACT applications, stem cells or immune cells are isolated from patients for precisely genome editing via the CRISPR/Cas9 system, and then the engineered cells are transplanted back into bodies. For instance, in cancer immunotherapy, the programmed death-1(PD-1) knockout in tumor-infiltrating lymphocytes (TILs) or chimeric antigen receptor T-lymphocytes (CAR-T) could dramatically elevate their cytotoxicity against cancer cells. Multiple clinical tests using this method are ongoing, such as two phase 1/2 trials of CTX110 and CTX120. The latest results of CTX110 gave a high complete response rate (CRR) of 50% indicating a promising future. The extension of ACTs in clinical studies could be mainly ascribed to the superior safety, where the edited cells can be scrutinized to guarantee editing efficiency and accuracy. The mature delivery strategies including electroporation and viral vectors have been constructed to transfer CRISPR/Cas9 nucleic acids or proteins into cell lines to produce the engineered cells. Moreover, the direct import of engineered cells avoids host immune responses associated with gene editing reagents [17]. In comparison, the in vivo genome editing therapies are more challenging due to the high requirements on tissue and cell selectivity, gene editing efficiency, and biosafety. Nevertheless, two clinical studies of EDIT-101 and CRISPR/Cas9-HPV16 E6/E7T1 have taken the first step in the in vivo genetic treatment. In EDIT-101, the photoreceptor cell-specific GRK1 promoter is used to precisely control the expression of Cas9, which could promise the cell selectivity of gene editing for minimized side effects after subretinal injection. The CRISPR/Cas9-HPV16 E6/E7T1 is formulated as hydrogel for topical administration and selective editing of cervical intraepithelial neoplasia. Collectively, the emerging clinical studies portend the splendid future of the CRISPR/Cas9 application in biomedicine. While the cell types amenable to isolation and in vitro editing are limited, since most post-mitotic and highly differentiated cells are manipulatable and functional only in vivo. The viral and non-viral vectors for in vivo CRISPR/Cas9-mediated genome editing undoubtedly have the potential to enable the treatment of various diseases by gene correction.

### 2.2. Physical Import

Physical import strategies are employed most frequently in clinical trials to deliver the CRISPR/Cas9 system into targeting cells, mainly including the electroporation and microinjection. The superiority of electroporation lies in that it can be applied in nearly all cell types at all stages of cell cycles, and is applicable to the delivery of molecules with a large hydrodynamic volume and even some nanoparticles. The temporary increase of cell membrane permeability during electroporation allows diverse types of CRISPR/Cas9 cargoes to translocate into the target cells with preferable efficiency and safety [18]. The electroporation has given promising performances in CRISPR/Cas9 delivery for therapeutic applications [19]. Nevertheless, the exposure of cells to a strong electric field would lead to significant cell death and loss of cell stemness. As a result, the electric field should be finely tuned to resist irreversible changes of cell viability.

In addition to electroporation, the microinjection is a straightforward physical strategy for CRISPR/Cas9 delivery using a micron-scale needle. Particularly, the microinjection could directly transfer macromolecules to the intended sites such as nucleus or cytoplasm, thereby offering a controlled manner to circumnavigate the associated delivery barriers. The microinjection has been successfully implemented in various cells with high efficiency, even up to 100%, and reduced the off-target effect from excessive editing due to the well-controlled quantity of injected CRISPR/Cas9 components [20]. The microinjection technology has been intensively employed to produce various knockout and transgenic animals for disease modeling [21]. The defect of microinjection is that every individual cell should be injected manually, making the strategy laborious in clinical applications.

### 2.3. Viral Vector Transfection

The viral vectormediated transduction of nucleic acid is another proven technique used in clinical trials for genome editing via the CRISPR/Cas9 system. Due to the superior transfection efficiency and wide applicability for various cells, the viral vectors have the potential for in vivo editing applications. For safety concerns, viral vectors are engineered to reserve transfection ability, while they would never replicate themselves or spread to other new cells. The first in vivo clinical trial of CRISPR/Cas9 has gained regulatory approval (NCT03872479), in which the adeno-associated virus (AAV) carrying CRISPR/Cas9 system was employed to correct CEP290 mutations in photoreceptor cells. The success of AAV has validated the great promise of viral vectors and paved the way for in vivo genetic manipulation.

Typical viral vectors for the CRISPR/Cas9 system are summarized in Table 2, including adenovirus (AV), lentivirus (LV), and AAV [22]. AAV is the most preferable choice due to its low immunogenicity, stable transgene expression, and serotype-related targeting. However, the synchronous encapsulation of Cas9 sequence, promoter, and sgRNA is challenging owing to the packaging limitation of AAV (around 4.5 kb). This issue can be partially solved using truncated SpCas9, SpCas9 fragments or smaller Cas9 orthologs such as *S. aureus* Cas9 (SaCas9) [23]. For instance, Villiger [24] exploited an intein-split base editor system that allows the splitting of the fusion nuclease into two parts, thereby overcoming the cargo limitation of AAVs. After intravenous injection, the AAV-base editor system led to significant gene correction rates of adult phenylalanine hydroxylase and restored physiological phenylalanine levels. The findings suggested the feasibility and high efficiency of AAV-mediated delivery. Nelson et al. [25] firstly tested the long-term effect of CRISPR/Cas9 genetic therapy in Duchenne muscular dystrophy (DMD). They found that the treatment on neonatal mice rather than adult mice could avoid AAV-associated immune responses, and dystrophin protein restoration induced by CRISPR-Cas9 could sustain for more than 1 year after a single intravenous administration. Moreover, the clinical application of AAV vector is limited by a pre-existing immunity against AAV, which would lead to invalidation of transfection via antibody neutralization [26].

Lentivirus (LV) is a single-stranded RNA spherical virus and the LV vectors possess many prominent merits including relatively mild immunogenicity, stable expression, high package capacity (8 kb), and excellent infection efficiency even in nondividing cells. Nevertheless, the LV vectors tend to induce insertional mutagenesis and the sustained Cas9 and sgRNA expression via LV vectors might bring off-target effects. To achieve transient Cas9 expression, Merienne [27] constructed a self-inactivating Cas9 system. LV vectors delivered plasmids encoding two different sgRNA. One was complementary to the target gene and the other could disrupt the Cas9-coding gene. The negative feedback control design could prevent the repeated expression of Cas9. To tackle the potential risk of inherent insertional mutagenesis of LV, the integration-defective LV (IDLV) was developed by point mutation in the integrase protein of LV [28]. However, the IDLV shows lower transgene expression as compared to their integrating counterpart. Sabina et al. improved the expression levels of IDLV for 6- to 7-fold by inserting the IS2 element [29].

Adenovirus (AV) is a double-strand DNA virus with an icosahedral nucleocapsid, and transfection capacity of both nondividing and dividing cells. The high packaging capacity of AV enables synchronous delivery of Cas9 sequence and multiple sgRNA for multi-target genome editing. Moreover, the AV genome remains extrachromosomal rather than integrating into the host genome, which could reduce potential off-target caused by re-expression of Cas9 and sgRNA. Despite the fact that AVs were accompanied with immune toxicities occasionally, they have been commonly used for delivering gene editing tools in mice. Xu [30] constructed an AV-mediated CRISPR/Cas9 system and corrected the DMD gene in skeletal muscle. The system could remove mutant exons via NHEJ mechanism and induced upregulation of dystrophin protein to about 50% of normal. Koo [31] co-delivered plasmids coding Cas9 protein and epidermal growth factor receptor (EGFR) mutation-specific sgRNA by AV to treat the EGFR-mutated lung cancer. Eventually, the mutant allele disruption significantly induced tumor apoptosis and restrained the growth of xenograft mouse tumor. Apart from disease treatments, the CRISPR/Cas9-loaded AVs have also been utilized for establishing disease models.

**Table 2:** Comparison of the viral vectors in clinical trials: AAV, LV, and AV.

Vector Type	Package Limitation	Superiority	Deficiency	References
AAV	4.5 kb	low immunogenicity, serotype-related targeting, stable transgene expression	low packaging capacity	[32]
LV	8 kb	large packaging capacity, low cell cycle tendency	long lasting expression of Cas9	[33]
AV	>8 kb	large packaging capacity, no-integration to host genome	high immunogenicity	[17]

### 2.4. The Obstacles of CRISPR/Cas9 Genome Therapy In Vivo

As a proven technique, the viral vectors hold vast potential for in vivo genome therapy due to their general applicability and superior transfection efficiency [33]. In the clinical investigation of EDIT-101, adeno-associated virus-5 is used as the vector for the CRISPR/Cas9 system, with photoreceptor cell-specific GRK1 promoter constructed in the viral vector for precise control of Cas9 expression. The promoter-control design could effectively enhance the cell-selectivity to circumvent the off-target effect after subretinal injection. The strategy could be applicable in the topical administration applications of the viral vectors. However, we suppose that the benefits of the strategy would be reduced in systematic administration due to the insufficient targeting ability of the viral vectors. Given the underlying immunogenicity, carcinogenesis, and insertional mutagenesis of viral vectors, developing biocompatible non-viral vectors with comparable delivering efficiency represents a promising strategy for in vivo genome editing. Rationally designed nanovectors could give multi-functional performances of lower off-target risks, remarkable packaging capacity, programmable targeting route, negligible immunogenicity, and spatiotemporal control of editing.

## 3. Non-Viral Nanovectors for CRISPR/Cas9 Delivery

**Figure 2:**
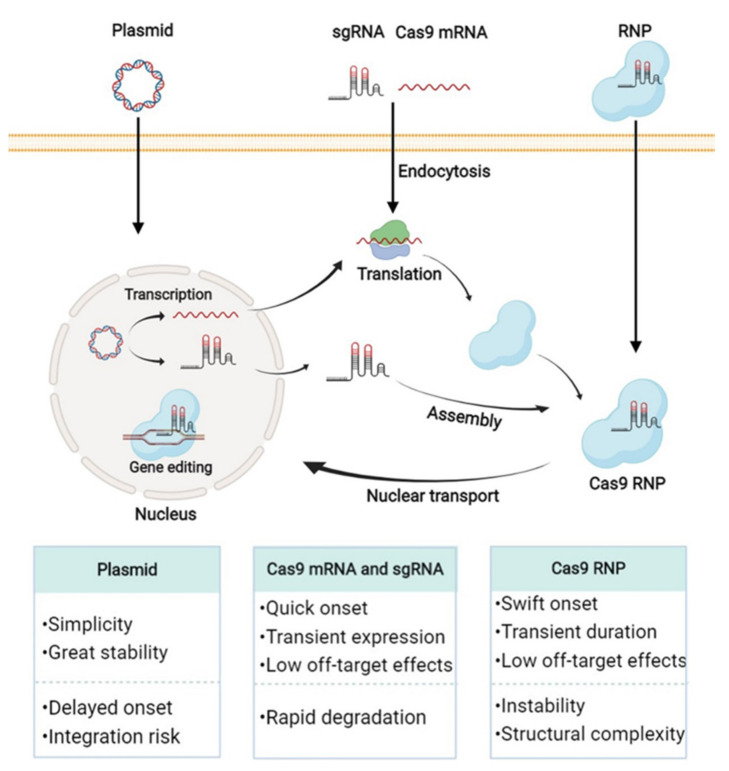
Representative genome editing routes and features of CRISPR-Cas9 in the form of DNA, RNA, and RNP (Created with BioRender.com, accessed on 29 September 2021).

The defects of physical and viral approaches such as cell injury, limited packaging capacity, and immune activation, have promoted the progress in non-viral nanovectors. With the tunable structure for various engineered biofunctions, the synthetic and endogenous material constructed nanovectors are likely to play a predominant role in the near future. Typically, three forms of CRISPR/Cas9 system are available for delivery including plasmid DNA (pDNA), RNA system of Cas9 mRNA and sgRNA, and Cas9 RNP (Figure 2).

**Figure 3:**
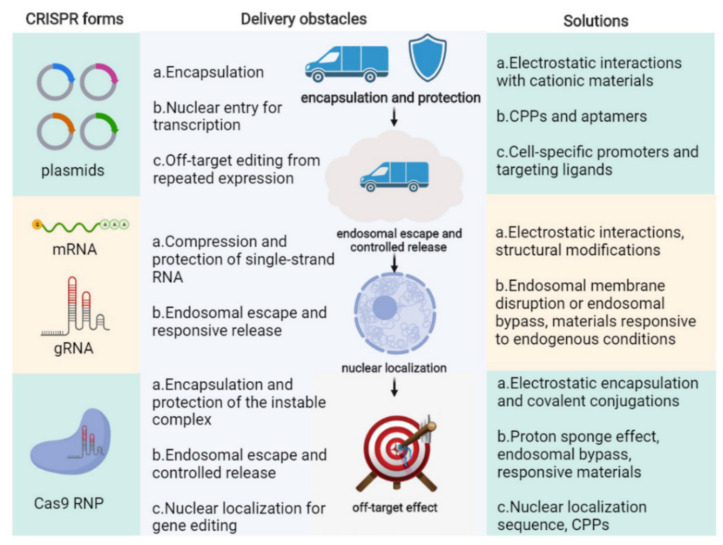
The underlying obstacles and corresponding solutions for efficient CRISPR system delivery in the form of plasmid, RNA (Cas9 mRNA, sgRNA), and Cas9 RNP (Created with BioRender.com, accessed on 30 September 2021).

The original CRISPR/Cas9 plasmid for gene editing in eukaryotic cells was constructed by Le et al. and termed as pX330 [5]. Two expression cassettes are contained in the pX330, where the U6 promoter is used to drive gRNA expression in one cassette, and a chicken β-actin promoter is used to drive SpCas9 expression in another. Particularly, the promoter for Cas9 in the plasmid could be substituted with a cell-specific promoter for the selective expression of Cas9 in target cells. Moreover, the nuclear localization signal (NLS) similar to SV40 NLS is required for transporting the CRISPR/Cas9 system into the nucleus, which could be fused with Cas9 [34]. The Cas9 protein possesses the molecular weight of 160 kDa and gene length around 4 kb, with a hydrodynamic diameter of 7.5 nm and positive surface charge, leading its problematic delivery. Two forms of gRNA are available for genome editing: A customized CRISPR RNA (crRNA) together with a common transactivating CRISPR RNA (tracrRNA) or a single guide RNA (sgRNA) consisting of 20 nucleotides targeting the RNP complex to the DNA, and a backbone sequence anchoring it to the Cas9. The sgRNA is approximately 100 bp, with a hydrodynamic diameter of 5.5 nm and negative charge [35]. The sgRNA suffers from rapid degradation, which could be relieved by chemical modifications including internucleotide linkage modification, sugar modification, and nucleobase modification. As shown in Figure 2 and Figure 3 due to the different pathways of action and intrinsic properties of the three forms, the delivery of each form is faced with different obstacles. Compared with the mRNA system and Cas9 RNP, the transfection of CRISPR/Cas9 pDNA stands out due to the preferable stability and cost-effectiveness. Nevertheless, the delivery concerns of compression, nuclear location, and off-target risks from the repeated expression have to be overcome for effective pDNA-mediated genome editing. In the case of RNA system delivery, the nanovectors ought to be capable of the simultaneous transport and protection of the relatively unstable Cas9 mRNA and sgRNA, as well as the controllable release in cytoplasm. Moreover, the transfection of Cas9 RNP could skip the expression of Cas9 protein and sgRNA for direct genome editing with notably lower off-target risks, but the efficient delivery of Cas9 RNP remains a serious challenge due to its large molecular size, instability, and low efficiency of endosomal escape.

### 3.1. CRISPR/Cas9 Plasmid Delivery with Nanovectors for Compression, Nucleus Targeting, and Off-Target Reduction

The plasmid-based CRISPR/Cas9 is an appealing approach due to its simplicity, stability, and low cost of manipulation. The plasmid could also facilely realize the multiplexed gene editing synchronously through the design of multiple sgRNA targeting different genomic locations. Theoretically, the delivery of the Cas9 plasmid using non-viral vectors should follow the similar primary principles as for other plasmids. The nanovectors could be formulated by electrostatic interactions and further optimized for small size, maximal cellular uptake, protection from degradation, and opportune intracellular liberation. Differently, the Cas9 and sgRNA expression plasmids with a structure larger than 10 kb is difficult to compress into nanoparticles and translocate to the nuclei [4]. In addition, the repeated expression of the Cas9 and sgRNA would increase the off-target risks, which could be relieved by the delivery strategies.

#### 3.1.1. Delivery Systems for Effective Compression

In general, the SpCas9 expression cassette of longer than 4 kb was constructed in one plasmid together with sgRNA cassette, reporter genes, and HDR template, generating a large CRISPR/Cas9 plasmid that is hard for encapsulation. The effective compression of CRISPR/Cas9 plasmids contributes to the transfection improvement. Zhang et al. [36] screened more than 56 delivery candidates, most of which demonstrated a limited efficiency due to the deficient package of the plasmids. Thereafter, they proposed an optimized novel polyethylene glycol (PEG) phospholipid modified cationic lipid nanoparticle to package Cas9 plasmids, generating a core-shell structure. The negative charged compact core was composed of Cas9/sgRNA-fused plasmids, chondroitin sulfate, and protamine, where the chondroitin sulfate functioned to enhance the condensation of the plasmids by electrostatic interactions. The cationic lipid shell was further used to encapsulate the negative charged core to promote the transfection. The core-shell structured nanoparticles-mediated 47.4% transfection of the plasmid in A375 cells in vitro and contributed to the notable downregulation of Polo-like kinase 1 protein and the corresponding tumor suppression over 67% in vivo.

Exosomes are natural particles with high biocompatibility and negligible immunogenicity owing to the endogenous bio-structure [37]. With the merits of multi-drug loading capacity, biostability in circulation, escape from phagocytosis of mononuclear phagocytes, biological barriers crossing, and various targeting abilities, the exosomes are potential vectors for CRISPR/Cas9 plasmids. Kim et al. [38] constructed cancer-derived exosomes as natural carriers for tumor-targeted CRISPR/Cas9 plasmid delivery and circumvention of underlying immunogenicity and toxicity of cationic materials. The exosomes isolated from human embryonic kidney 293 cells (HEK293) and human ovarian cancer cells (SKOV3) shared superior ovarian tumor selective accumulation compared with epithelial cell-derived exosomes. The CRISPR/Cas9-containing exosomes could suppress the expression of poly (ADP-ribose) polymerase-1 (PARP-1) to induce the tumor apoptosis and enhance the chemosensitivity of tumor cells to cisplatin. Despite the promise in CRISPR/Cas9 delivery, the biomedical applications of exosomes were hindered by the limited packing capacity. To address the issue, Lin [39] firstly established a hybrid nanoparticle via incubating original exosomes with Lipofectamine 2000. The generating exosome-liposome nanoparticle integrated the high DNA compression with the exosome biofunctions, which could be endocytosed by Lipofectamine 2000-resistant mesenchymal stem cells (MSCs). The hybrid nanoparticle could mediate the effective gene editing in MSCs and HEK293FT cells without obvious toxicity.

**Figure 4:**
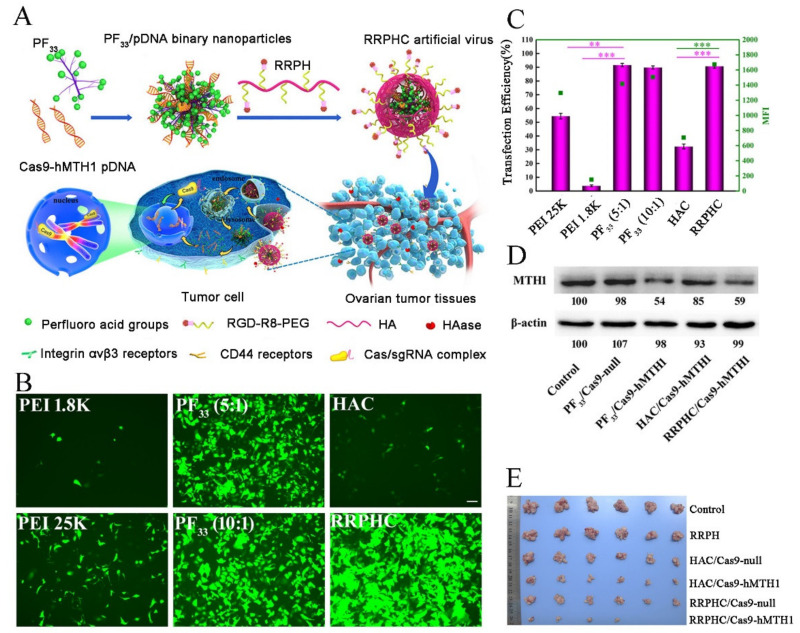
Fluorinated PEI-based artificial virus for Cas9 plasmids delivery. (**A**) Schematic illustration of the multifunctional artificial virus. (**B**) Transfection efficiency of different nanoparticles loading EGFP plasmids at 24 h. The images were taken by fluorescence microscopy in SKOV3 cancer cells. (**C**) Quantitative analysis of transfection efficiency in SKOV3 cells. (**D**) Western-blotting assays reflecting the expression level of MTH1 protein. (**E**) Representative photos of ovarian cancer cells after the in vivo treatment. Significant differences between groups were indicated as ** *p* < 0.01, and *** *p* < 0.001. Reproduced with permission from [40]. Copyright ACS Publications, 2017.

Over the past decades, the cationic polymer such as polyethylenimine (PEI), chitosan, and poly(L-lysine) were identified as efficient nucleic acid carriers. Notably, the amino-abundant PEI could readily compress the anionic plasmids into a compact nanostructure, with superior proton-sponge effect for endosomal escape, making it a “gold standard”. The branched PEI of 25 kDa was verified to deliver CRISPR/Cas9 plasmids into mouse neuroblastoma Neuro2a cells with high efficiency, and meditate genome editing within the targeted locus. Nonetheless, the potential cytotoxicity of PEI polymers is non-ignorable, which could be alleviated by fluorinated group modification, accompanied with enhanced transfection efficiency. Li et al. [40] constructed a multifunctional nucleus-targeting “core-shell” nanovector to transport CRISPR/Cas9 plasmids. As shown in Figure 4A, the plasmids were compacted with a PEI-derived fluorinated polymer (PF33), followed by the construction of a versatile multifunctional shell (RGD-R8-PEG-HA, RRPH). The PF33 core could promote endosomal escape for the enhanced transfection, while the RRPH shell endowed the nanovector with biostability, multiple tumor targeting, and deep tumor penetrating. The core-shell nanovector gave a high transfection over 90% in SKOV3 cells (Figure 4B,C), and enabled the efficient downregulation of MTH1 protein by the CRISPR/Cas9 system (Figure 4D). Moreover, the in vivo evaluation suggested the disruption of MTH1 and significant tumor suppression (Figure 4E).

#### 3.1.2. Delivery Systems for Guiding Plasmids into the Nucleus

The transcription of plasmids into the nucleus is the primary step for the genome editing action of plasmid-based CRISPR/Cas9 system. Therefore, the nuclear translocation of the CRISPR/Cas9 is supposed to benefit the genome editing, while the supramaximal plasmid with an intensive negative charge could hardly pass through the nuclear pore. The decoration of nuclear localization modules on nanovectors is a promising approach for overcoming the obstacle. The cell-penetrating peptide (CPP) is the preferable candidate for nuclear-localized modification owing to the superior cell membrane permeability. Wang et al. [41] condensed CRISPR/Cas9 plasmids on TAT peptide-decorated gold nanoparticles (AuNPs/CP) via electrostatic interactions, and then the plasmid-loaded AuNPs was encapsulated with cationic lipids to generate the LACP nanoparticles. The lipid shell contributed to the high biostability and cellular internalization of the nanoparticles. The CRISPR/Cas9 plasmids could be liberated into the cytosol via laser-triggered thermo-effects, followed by the nuclear translocation of CRISPR/Cas9 plasmids upon the TAT guidance. The LACP nanoparticles enabled the efficient knockout of Plk-1 gene in melanoma tumors and significant tumor inhibition both in vitro and in vivo.

Given that the nucleolin is highly expressed on the cell nuclei and membranes, the AS1411 aptamer with advanced affinity to nucleolin was expected to mediate the nuclear delivery of the cargoes. Liu et al. [42] constructed an AS1411-modified polymer/inorganic hybrid nanoparticle to edit the tumor cells with high efficiency. CRISPR/Cas9 plasmids were condensed into a nanocore with calcium carbonate, protamine sulfate, and calcium phosphate by coprecipitation, and then the surface was decorated by a carboxymethyl chitosan layer functionalized with biotin for tumor cell targeting and AS1411 for nuclear localization. The hybrid nanovector induced a sharp decrease of 90% in CDK11 protein expression.

#### 3.1.3. Delivery Systems for Reducing the Off-Target Effect by Enhanced Cell Selectivity

**Figure 5:**
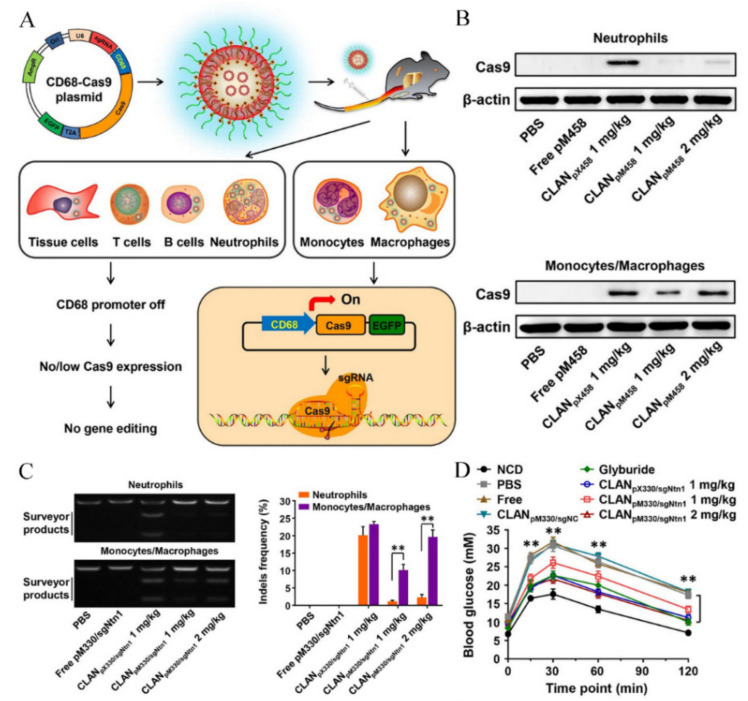
Macrophage-specific in vivo genome editing using CLAN. (**A**) Rational design of CLAN for introducing Cas9 expression plasmids into different cell types. Under the control of CD68 promoter, Cas9 was specifically expressed in macrophages and monocytes. BHEM-chol is a cationic lipid named *N*,*N*-bis(2-hydroxyethyl)-*N*-methyl-*N*-(2-cholesteryloxycarbonyl aminoethyl) ammonium bromide. (**B**) Cas9 protein expression in neutrophils and monocytes/macrophages 48 h after intravenous injection. These cells were isolated from peripheral blood, liver, spleen, and adipose tissue. (**C**) Detection of Ntn1 gene disruption efficacy with T7E1 assays after intravenous injection. Surveyor products cleaved by T7EI (left) and indels frequency (right) in the Ntn1 locus. Data are shown as the means ± SD (*n* = 5), ** *p* < 0.01. (**D**) Glucose tolerance tests. Reproduced with permission from [43]. Copyright ACS Publications, 2018.

In genetic therapy, the off-target concerns should be minimized for potential clinical applications. Due to the relative stability of plasmid, the Cas9 and sgRNA could be expressed repeatedly, leading to the increase of the off-target risks. Primarily, the rational design of the plasmid sequence could endow the plasmid with controllable and specific expression. Luo et al. [43] designed a macrophage-specific promoter-driven plasmid for macrophage-selective gene editing by the CRISPR/Cas9 system. As shown in Figure 5A, the classical chicken β-actin promoter of pX330 and pX458 was replaced by the macrophage-specific CD68 promoter to circumvent off-target editing in undesired cells. The engineered plasmids were encapsulated by a cationic lipid-assisted PEG-PLGA nanoparticle (CLAN) for effective transfection. After administration, the CD68 promoter could drive the specific expression of Cas9 (Figure 5B) and allow the effective disruption of netrin-1 gene, which is reported as a potential therapeutic target for type 2 diabetes (Figure 5C). The final in vivo evaluation showed improvements of insulin sensitivity and diabetes symptoms after treatment with CLAN-mediated genetic therapy (Figure 5D).

**Figure 6:**
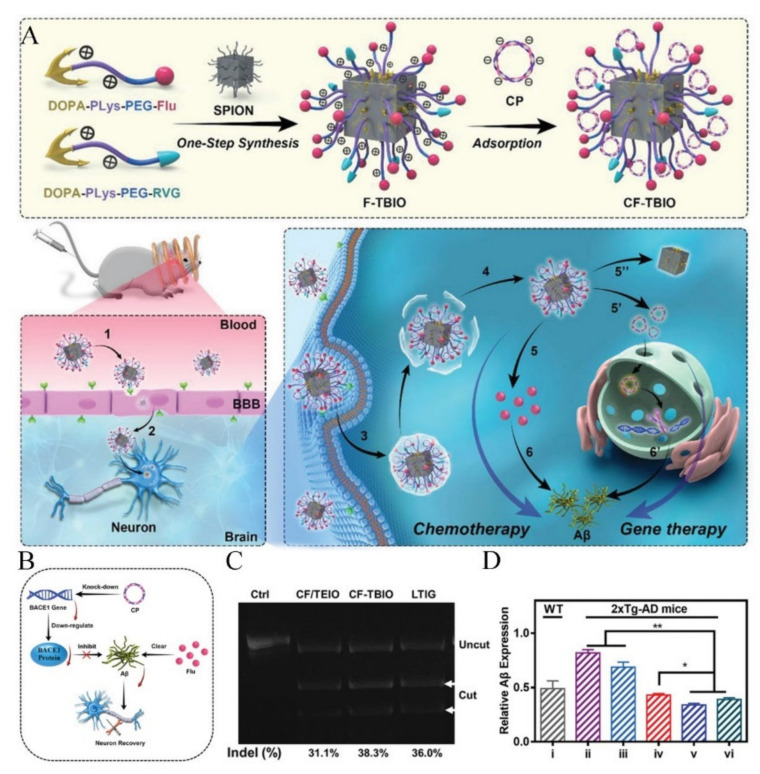
Illustration of CRISPR/Cas9 nano-biohybrid complexes for neurodegenerative diseases treatment. (**A**) Preparation of CF-TBIO and illustration of the therapeutic mechanism. CF-TBIO were injected through the tail vein. CF-TBIO nanoparticle crossed the BBB and targeted neurons with the guidance of RVG. The ester bonds were hydrolyzed by intracellular esterase and release Fluvastatin to clear Aβ. CRISPR/Cas9 plasmids then entered the nuclei and knocked out the BACE1 gene. (**B**) Diagram of CRISPR/Cas9 plasmids and Fluvastatin for AD treatment. (**C**) T7E1 assay for BACE1 indels frequency analysis in brains of double transgenic (2×Tg-AD) mice. (**D**) Quantification of WB analysis of Aβ expression. (i) Wild type mice without treatment, (ii) 2×Tg-AD mice without treatment, (iii) 2×Tg-AD mice treated with CNSF-TBIO, (iv) CF/TEIO, (v) CF-TBIO, (vi) the mice were administrated via the tail vein every 10 days, while the other groups were every 3 days. Significant differences between groups were indicated as * *p* < 0.05, and ** *p* < 0.01. Reproduced with permission from [44]. Copyright Joh Wiley and Sons, 2021.

The functionalization of nanovectors could provide alternative approaches to alleviating the off-target risks. The programmed delivery and targeting pathway could effectively reduce the off-target toxicity in normal cells. Recently, Shen et al. [44] designed a multi-functional nanovector for the co-delivery of CRISPR/Cas9 plasmids and Fluvastatin into brain lesions. As illustrated in Figure 6A, synthetic DOPA-Polylysine were coupled with Fluvastatin or rabies virus glycoproteins (RVG) through PEG, generating the long-chain bio-polycations (DOPA-PLys-PEG-Flu/RVG). Thereafter, the bio-polycations were anchored onto superparamagnetic iron oxide nanoparticles via DOPA. Eventually, the CRISPR/Cas9 plasmids were loaded on the biohybrid complexes by electrostatic interactions. The nanoparticles could cross the blood–brain barrier and internalize into neuronal cells specifically under the guidance of RVG ligand. The Fluvastatin could eliminate the existing amyloid-β (Aβ) and the subsequent genetic therapy contributed to the permanent downregulation of BCE1 gene and further affected the Aβ level (Figure 6B–D). The multi-functional nanovector shared advanced therapeutic benefits against the Alzheimer’s disease (AD), without causing obvious systemic side effects.

### 3.2. Cas9 mRNA and sgRNA Delivery with Nanovectors for Protection, Compression, and Controllable Release

The RNA-based CRISPR system (Cas9 mRNA and sgRNA) is a promising candidate for in vivo genome editing, due to the superiorities over the plasmid including smaller molecular structure, rapid onset of action, and relieved off-target effect from repeated Cas9 and sgRNA expression. Cas9 mRNA could be straightly translated into Cas9 protein after entering the cytoplasm, and then assemble into RNP with sgRNA followed by translocation into the cell nucleus for genome editing. Moreover, the transient expression of Cas9 nucleases could help precisely control the dosage of Cas9 proteins and reduce risks of the off-target effect. However, the challenge for Cas9 mRNA and sgRNA delivery is that the single-stranded structure is quite fragile compared with plasmids. Multiple chemical modifications have been employed to improve the resistance to RNases such as substitution with pseudouridine, N6-methyladenosine or inosine [45,46]. Alternatively, the rational designed nanovectors could protect RNA molecules from the degradation, and endow them with endosomal escape capacity and controllable liberation for magnified genome editing efficiency.

#### 3.2.1. Delivery Systems for mRNA and sgRNA Encapsulation and Protection

Given that the poor stability of RNA is a vital hurdle in the application of Cas9 mRNA and sgRNA, various delivery strategies are proposed for RNA protection during the systemic shuttling. Jonathan et al. [47] reported a lipid nanoparticle (LNP)-based carrier termed as LNP-INT01 that could co-formulate Cas9 mRNA and sgRNA into a single nanoparticle. A novel biodegradable, ionizable lipid named ‘‘LP01′’ was synthesized for LNP-INT01 fabrication together with PEG-DMG lipids. The Cas9 mRNA and sgRNA were structurally modified for further stabilization in vivo. The LNP-based delivery platform for the Cas9 mRNA and sgRNA exhibited highly durable in vivo gene correction after a single, systematic administration. Qiang et al. proposed a strategy termed selective organ targeting (SORT), wherein the internal charge of LNPs was accurately tuned by changing the LNP molar compositions to enable tissue-specific gene editing [48].

In gene correction, the HDR mechanism could accurately manipulate the target sequence to form a predesigned sequence. However, three components of Cas9 protein, sgRNA, and DNA template ought to work cooperatively, which required the co-delivery of Cas9 mRNA, sgRNA, and donor ssDNA. Farbiak [49] engineered dendrimer-based lipid nanoparticles (dLNPs) to encapsulate and deliver multiple components for in vivo HDR correction. The ratios of individual LNP components including ionizable amino dendrimer lipid, cholesterol, amphipathic phospholipid, and PEG2000-DMG were systematically optimized to effectively compress the mixture of nucleic acids with different chemical structures. The dLNPs nanoplatform eventually accomplished 55% HDR efficiency in vitro and nearly 20% efficiency in xenograft tumors in vivo.

Since the extracellular vesicles (EVs) represent a natural mode of intercellular communication, the EVs-inspired vector for the RNA drug could provide high biostability and cellular uptake efficiency with negligible cytotoxicity. Usman et al. [50] developed the red blood cells (RBCs)-derived extracellular vesicles (RBCEVs) for the delivery of RNA drugs including Cas9 mRNA, and to guide RNAs, antisense oligonucleotides. The mRNA-based CRISPR/Cas9 system delivery with RBCEVs shared highly robust genome editing in both human cells and xenograft mouse models, with no observable cytotoxicity. Generally, the RNA molecules were encapsulated into the EVs by electroporation, which possessed a relatively poor loading efficiency. Li et al. constructed a fusion protein of membrane protein CD9 and human antigen R (HuR), which could anchor onto the EVs membrane. The HuR could bind to the AU-rich elements (AREs) in RNA molecules with a relatively high affinity for the enhanced loading of RNA cargoes. The decorated exosomes were confirmed to have considerable potency for AREs modified Cas9 RNA encapsulation [51].

The block copolymers could form the stable core-shell nanostructure for RNA compression and protection. Abbasi et al. [52] proposed a PEGylated polycation block copolymer (PC) to enable the stabilization of RNA-based CRISPR/Cas9 system during the in vivo delivery. The copolymer consisted of two parts, including PEG and poly(N′-(N-(2-aminoethyl)-2-aminoethyl) aspartamide (PAsp). The amino-rich PAsp could compress the negative charged RNA by electrostatic interaction, and facilitate the endosomal escape. In addition, the spontaneously-formed PEG corona could protect RNA molecules from enzymolysis and reticuloendothelial system (RES) elimination for long circulation and brain tissue diffusion improvement. Consequently, the PC-mediated RNA delivery induced efficient gene editing in brain parenchymal cells.

#### 3.2.2. Delivery Systems for RNA Responsive Release

**Figure 7:**
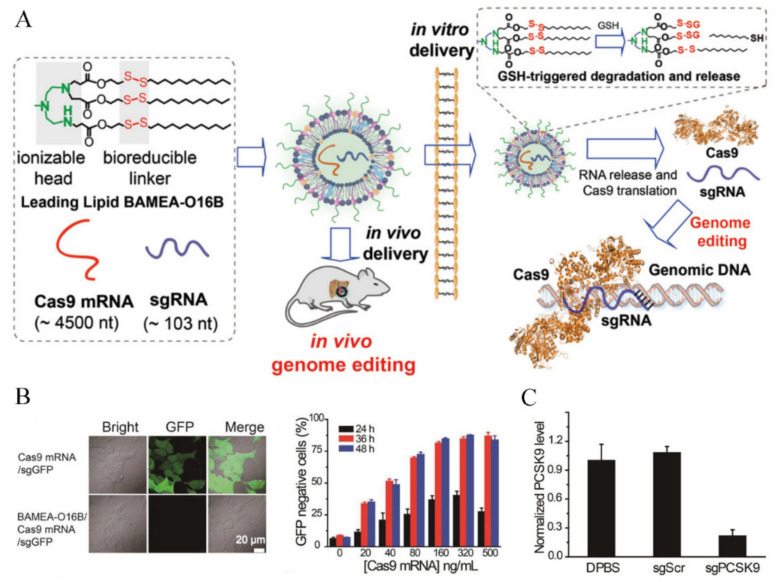
Fast and efficient Cas9 mRNA and sgRNA delivery by bioreducible lipid nanoparticles. (**A**) Schematic diagram of BAMEA-O16B lipid nanoparticle for Cas9 mRNA and sgRNA delivery. (**B**) CLSM images and quantitative analysis of GFP expression of HEK-GFP cells. BAMEA-O16B nanoparticles showed complete loss of GFP fluorescence, while Cas9 mRNA/sgGFP alone did not show a comparable outcome. GFP knockout efficiency can increase to higher than 90%. (**C**) The mouse serum PCSK9 level decreased to 20% of control group after intravenous injection of BAMEA-O16B nanoparticles. Reproduced with permission from [54]. Copyright Joh Wiley and Sons, 2019.

The intracellular liberation of RNA payloads from the vectors is the prerequisite for effective transfection and genome editing. The bio-stimuli triggered degradation of the nanovectors is a potential strategy for rapid RNA molecule release [53]. Liu et al. [54] reported a reducible lipid nanoparticle (BAMEA-O16B) to encapsulate Cas9 mRNA and sgRNA for swift and effective genome editing (Figure 7A). BAMEA-O16B could load CRISPR RNAs by electrostatic interaction and liberate the payloads rapidly in cells via the reduction of disulfide bonds. The CRISPR RNAs released in cytoplasm could effectively knock down the expression of GFP in human embryonic kidney (HEK) cells (Figure 7B). Notably, the rapid gene knockout was observed at 24 h post RNA delivery, and the in vitro knockout efficiency was higher than 90%. To evaluate the in vivo genome editing potency, the reducible RNA delivery system was injected intravenously to knockout the PSCK9 gene in mouse hepatocytes, a valid therapeutic target for cardiovascular diseases. The PSCK9 level in mouse serum decreased to 20% of the control group (Figure 7C), confirming the enhanced genome editing by the design of bio-stimuli triggered RNA molecule liberation.

### 3.3. Cas9 Ribonucleoprotein Delivery with Nanovectors for Package, Controllable Liberation, and Nuclear Localization

The Cas9 ribonucleoprotein (RNP)-based CRISPR/Cas9 system could give the swiftest gene editing via a skip of the expression of protein and sgRNA in cells compared with the plasmid and RNA forms of CRISPR/Cas9. Moreover, the Cas9 RNP transfection could avoid the DNA integration into the genome, and minimize the off-target risks caused by the repeated expression of CRIPSR system. Although the merits of Cas9 RNP delivery would improve the gene editing, the efficient delivery of Cas9 RNP remains a challenge. The Cas9 protein and sgRNA are inherently instable and the large size and the low efficiency of endosomal escape of Cas9 RNP would also hinder its efficient delivery. The principles and features of delivery strategies for Cas9 RNP are discussed to overcome the obstacles.

#### 3.3.1. Encapsulation and Protection of Cas9 RNP

The efficient package and stabilization of Cas9 RNP by the carriers is the prerequisite for delivery applications. Owing to the net negative charge of Cas9 RNP, cationic polymers and liposomal components are commonly used for the Cas9 RNP package via electrostatic attraction. For instance, in situ polymerization of charged monomers has been confirmed as a flexible method to encapsulate Cas9 RNP. Chen et al. [55] reported a customizable nanocapsule to package preassembled Cas9 RNP, with various advantages of a small nanoparticle size (25 nm), high Cas9 RNP loading efficiency, as well as controllable stoichiometry and amenability to surface modifications. Due to the heterogeneous surface charge distribution of Cas9 RNP, the cationic and anionic monomers were employed to form a coating layer firstly by electrostatic interaction. Then, the GSH-degradable crosslinker, mPEG, PEG conjugated with ligands, and imidazole-containing monomers were absorbed to the surface via hydrogen bonding and Van der Waals interactions for the subsequent in situ free-radical polymerization to generate the nanocapsule. After orthotopic injection, robust gene editing was detected in skeletal muscle and murine retinal pigment epithelium (RPE) tissue.

Polymers have been extensively used for delivering nucleic acids and proteins due to the merits of flexible structures, ease of synthesis, and facile functionalization. For polymer-mediated protein delivery, the effective encapsulation is the major obstacle. Normally, the protein molecules with different isoelectric points may be positively or negatively charged and make it difficult to design a universal polymer. To address this concern, a protein supercharging strategy was proposed to increase binding sites on proteins for cationic polymers. Cationic proteins were modified with anionic species such as anionic proteins and peptides, anionic polymers, carboxyl-rich chemicals, and nucleic acids. For instance, the negatively charged RNP complex enables the positive-charged Cas9 protein to form more stable nanoparticles with cationic materials. In addition to the protein supercharging strategy, Liu et al. [56] proposed a boronic acid-rich dendrimer with excellent delivery efficiency of various native proteins, which could obviate the chemical modification of proteins. The phenylboronic acid (PBA)-rich polymer could package proteins of different charges via a combination of cation-π, nitrogen-boronate complexation, and ionic interactions. The dendrimer could assemble with various proteins to form nanoparticles, accompanied with well-maintained bioactivities. The PBA-rich polymer was also suitable for the effective delivery of Cas9 RNP. In addition, the significant decrease of EGFP expression gene in the EGFP stably transfected human embryonic kidney 293T (293T-EGFP) cells further confirmed the enhanced genome editing.

Cationic polymers can also be applied to reverse the surface charge of Cas9 RNP for encapsulation in anionic nanovectors. Cho et al. [57] prepared a lecithin-based liposomal vehicle for liver disease treatment via CRIPSR/Cas9-mediated genome editing. In order to enhance the encapsulation efficiency, cationic polymer PEI was fused with Cas9 RNP and formed a charge-reversed complex. The negatively charged lipids could wrap the complex by electrostatic interactions to generate a liposomal nanoparticle with uniform size distribution. Thereafter, the nanoparticle was applied to edit dipeptidyl peptidase-4 gene (DPP-4) in liver tissues for type 2 diabetes treatment. The expression of DPP-4 gene was disrupted in type 2 diabetes mellitus (T2DM) db/db mice with remarkable efficacy. The DPP-4 enzyme activity decline contributed normalized blood glucose levels, insulin response, and reduced liver and kidney damage.

**Figure 8:**
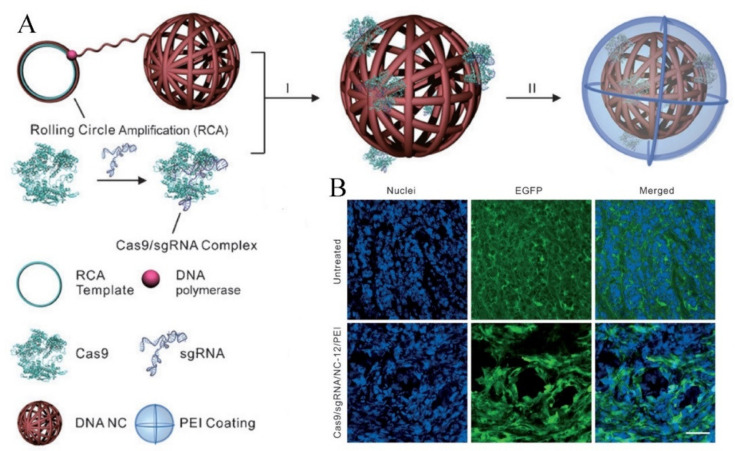
Self-assembled DNA nanoclew for CRISPR/Cas9 RNP delivery. (**A**) Design of the DNA nanoclew to deliver the CRISPR-Cas9 RNP complex. (**B**) In vivo gene editing of Cas9 RNP in U2OS. EGFP xenograft tumors. The EGFP stained by antibodies appears green, and the nuclei appears blue. Approximately 25% of the tumor cells near the injection site showed no EGFP expression and the untreated mice did not show any loss. Reproduced with permission from [58]. Copyright John Wiley and Sons, 2015.

Apart from the electronic interaction, the base-pairing ability of sgRNA was also harnessed for Cas9 RNP encapsulation. In the structure of Cas9 RNP, the negative-charged sgRNA is loaded into the groove of a positive-charged nuclease (NUC) lobe and an α-helical recognition (REC) lobe. The single stranded sgRNA could serve as a bridge for the encapsulation of Cas9 RNP. Vectors with a properly designed nucleic acid sequence could be linked to the conservative region in sgRNA through complementary base pairing. DNA nanostructures represent a promising delivery platform with merits of various drug loading capacity, biodegradability, and biocompatibility. As shown in Figure 8A, Sun et al. reported a biologically inspired DNA nanoclew synthesized by the rolling circle amplification (RCA) [58]. The DNA nanoclew was composed of palindromic sequences partially complementary to sgRNA, which enabled Cas9 RNP to load onto its surface via base pairing. Thereafter, the PEI polymer was applied to fabricating the corona for enhanced cellular uptake and endosomal escape. The nuclear-localization-signal peptides fused on the Cas9 protein could guide the translocation of Cas9 RNP into the nucleus for effective genome editing. The transfection results confirmed that the RNP loaded DNA nanoclew could effectively knockout the EGFP gene (Figure 8B).

The thiol-terminated DNA sequences complementary to sgRNA are readily reactive with gold nanoparticles (AuNPs) and anchored on the AuNPs surface, which enables the encapsulation of Cas9 RNP by base-pairing. Lee et al. [59] established a “CRISPR-Gold” vehicle composed of donor DNA, Cas9 RNP, ssDNA-conjugated gold nanospheres, as well as silica and cationic polymers, which aimed to realize HDR-mediated gene repair. In fabrication, ssDNA-conjugated AuNPs were selected as a core, followed by the donor DNA hybridization with ssDNA. The RNP was subsequently absorbed onto the AuNPs core via base-pairing affinity between sgRNA and donor DNA. Thereafter, the particles were encapsulated by silica and polyaspartic acid for stabilization and endosomal disruption via the proton sponge effect. The glutathione in the cytoplasm could trigger the rapid release of donor DNA and RNP for gene repair. The “CRISPR-Gold” nanovector could effectively repair the mutated dystrophin gene in mice by inducing HDR in muscle tissue. Furthermore, the “CRISPR-Gold” nanovector was proved to deliver the Cas9 RNP and donor DNA to major brain cells. The expression of metabotropic glutamate receptor 5 (mGluR5) gene was significantly suppressed after intracranial injection. The results suggested that the “CRISPR-Gold” has great potential to treat neurological diseases such as autism, and facilitate the development of animal models for these diseases [60].

Moreover, the covalent modification of Cas9 protein is an effective strategy for Cas9 RNP encapsulation and protection. The cell-penetrating peptides (CPPs) are short cationic polypeptides with 10-30 amino acids in length, which could covalently conjugate to the Cas9 protein to enable the delivery of cargoes into the cytosol through passive or active endocytic pathways. For example, the Cas9 RNP fused with a supercharged peptide called SCP could be efficiently internalized into the target cells, followed by an escape from the endosomes, and translocation into the nucleus [61]. However, the covalent strategies have underlying risks of structure alteration and bioactivity reduction. Gustafsson [62] repurposed the PepFect14 (PF14) peptide, an amphipathic cell-penetrating peptide commonly applied in RNA delivery, to improve the transport of Cas9 RNP. Previous attempts via covalent conjugation achieved low editing efficiency, since the covalent conjugation of CPPs may interfere with Cas9 complexation to the sgRNA. In consequence, the PF14 peptide was used to form a complex with Cas9 RNP via ionic interaction, and the complex demonstrated high editing rates up to 80% in HEK293T cells without any apparent toxicity. Branched polymers with low molecular weight could also be conjugated on the Cas9 protein for delivery applications. Yoo et al. [63] constructed a branched polyethylenimine (bPEI, Mw 2000 Da) functionalized Cas9, which could form a complex with sgRNA and donor DNA. The complex shared a greatly enhanced internalization into the cells compared to the native Cas9 RNP and efficient gene correction by the homology-directed repair. Alternatively, the branched PEG was employed for Cas9 modification and functioned as a linkage between Cas9 and asialoglycoprotein receptor ligands. The modified RNP could be preferentially internalized into cells expressing the corresponding receptor on their surface. The receptor-mediated delivery of genome-editing enzymes provides an approach for cell-selective gene editing [64].

#### 3.3.2. Bio-Responsive Nanoparticles for Endosomal Escape and Controlled Release

**Figure 9:**
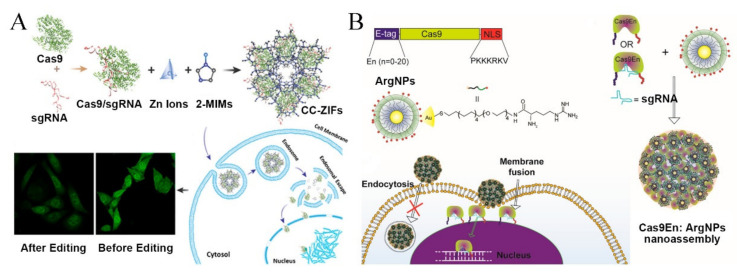
Responsive nanoparticles for endosomal membrane disruption and cell membrane fusion. (**A**) Preparation, cytoplasmic delivery, and gene editing efficiency of Cas9 RNP/ZIF (Reproduced with permission from [66]. Copyright ACS Publications, 2018). (**B**) Rational engineering of Cas9 protein and ArgNPs for intracellular delivery of Cas9-RNP via membrane fusion (Reproduced with permission from [69]. Copyright ACS Publications, 2017).

Most of the nanovectors are transported following the endosome-lysosome pathway, which was liable to cause the degradation of Cas9 RNP. Therefore, the protection of RNP cargoes and swift escape from endosomes is of great importance for the transfection. The proton sponge effect is the most widely recognized mechanism to promote the disruption of endosomal membranes [65]. When trapped in endosomes, the abundant amine groups were protonated, which affected the H^+^ and Cl^−^ pumping and prompted water flowing into endosomes, leading to the swelling and disruption of the endosomes. For instance, Alsaiari et al. [66] firstly delivered Cas9 RNP by a metal-organic material called zeolitic imidazole frameworks (ZIFs). ZIFs consisted of imidazolate linkers and tetrahedrally-coordinated transition metal ions with tunable pore openings to load the Cas9 RNP. The Cas9 RNP protein could interact with Zn^2+^ ions in ZIFs via coordination and ionic interactions, and the imidazolate linkers gave an excellent pH-buffering capacity and a corresponding ability of the endosomal escape. This system was verified as suitable for transient gene editing and induced 37% suppression of green fluorescent protein (Figure 9A).

After the endosomal escape, the liberation of the Cas9 RNP is the prerequisite to initiate the genome editing. The rational-designed nanovectors that could respond to the various microenvironments during the transport are supposed to elevate the genome editing efficiency [67]. Guo et al. [68] designed a series of poly(disulfide)s to deliver different CRISPR/Cas9 forms including Cas9 RNP. Two functional monomers were synthesized, including Monomer 1 (M1) containing diethylenetriamine (DET) moieties, and Monomer 2 (M2) containing guanidyl ligands (CPD). The cationic CPD in M2 could form multiple hydrogen bonds and salt bridges with oxyanions in Cas9 proteins, and the primary amines of M1 would interact with the carboxyl groups of Cas9 protein for the encapsulation of Cas9 RNP cargoes. The protonated amines and guanidyl ligands could induce the endosomal escape. Thereafter, the disulfide linkers were readily cleavable by intracellular glutathione and triggered the rapid release of Cas9 RNP. The responsive degradation of polymeric vectors not only facilitated CRISPR/Cas9 genome editing, but also minimized the cytotoxicity caused by the accumulation of polymers.

Notably, the membrane fusion strategy could bypass the endosome-lysosome pathway. As illustrated in Figure 9B, Mout et al. [69] developed a remarkably high efficient direct cytoplasmic delivery vector of Cas9 RNP. The Cas9 protein was modified with an N-terminus glutamate peptide tag (E-tag) for electrostatic interaction with the cationic arginine-functionalized gold AuNPs and the fusion of nanoparticles to the cell membrane. The direct cytoplasmic delivery vector achieved high delivery efficiency of nearly 90% in various cell types.

#### 3.3.3. RNP Delivery Designs for Nuclear Localization

Nuclear-targeting delivery of Cas9 RNP is preferable for efficient intracellular genome editing as it promotes the transport of CRISPR components to the desired loci. The conjugation of nuclear localization sequence (NLS) at the end of Cas9 protein was commonly used to guide the nuclear translocation of Cas9 RNP. Moreover, the CPPs could facilitate the delivery of cargoes into the nucleus through passive or active pathways. Apart from traditional NLS and CPPs, the low molecular weight protamine (LMWP) demonstrated a remarkable membrane penetrating capacity. Kim et al. [70] developed a carrier-free delivery system by fusing Cas9 with LMWP. The Cas9-LMWP fusion protein carried both a NLS sequence and a positively charged LMWP for nucleus translocation. The positive-charged LMWP allowed the self-assembly of Cas9 protein with crRNA and tracrRNA via electrostatic interactions. LMWP attached to Cas9 could also function as a CPP with excellent endocytosis capacity and is less toxic than the traditional TAT peptides. The simple structure and nuclear-targeting ability made the ternary Cas9 RNP system an effective, safe, and precise genome editing system. The results demonstrated that the local injection of the ternary Cas9 RNP system effectively inhibited the A549 tumor growth via the disruption of KRAS gene.

## 4. Concluding Remarks and Future Perspectives

CRISPR/Cas9 has gained rapid development in recent years, providing an adaptable and accessible tool for genome manipulation and visualization. CRISPR/Cas9 holds tremendous potential as a therapeutic for diverse diseases related to genetic disorders such as β-thalassaemia, tyrosinemia, and cancers. The CRISPR/Cas9-based therapies have been evaluated in clinical trials. To date, most of these trials are focused on adoptive cell therapies, in which the target cells ought to be readily accessible. The delivery of CRISPR/Cas9 system in clinical trials mainly depends on physical approaches and viral vectors, which are seriously hampered by cell injury and safety concerns. The development of non-viral nanocarriers would contribute to extending the medical applications of CRISPR/Cas9 system.

The effective and safe delivery strategy remains a primary challenge for clinical applications of CRISPR/Cas9 system. Typically, three modes of CRISPR/Cas9 system are available for delivery: DNA plasmids, mRNA/sgRNA, and Cas9 RNP. The vectors should be rationally designed according to the action mechanism and physicochemical properties of each CRISPR/Cas9 mode for maximized genome editing efficiency, and minimized off-target risks. To date, various delivery systems for different CRISPR/Cas9 forms were developed including lipids, polymers, gold nanoparticles, DNA nanoclew, and cell penetrating peptides. Thereinto, the cationic lipid-based CRISPR/Cas9 plasmid delivery is the most commonly used strategy due to the mature technology of lipid nanoparticles and the structural simplicity, natural biostability, sequence editability, and compressibility of the plasmid. Nevertheless, the unavoidable drawbacks of delayed onset and integration risk drives the development of alternative approaches. The ideal strategy is supposed to be the direct delivery of Cas9 RNP into the nucleus, which could circumvent the expression of Cas9/sgRNA components for transient function, high genome-editing efficiency, and minimum off-target effect. The direct Cas9 RNP delivery strategy requires the smart vectors with multiple functions of Cas9 RNP encapsulation, internalization, endosomal escape, nuclear trafficking, and payload liberation. This represents a fertile research direction that can advance the CRISPR/Cas9 technology.

Despite the extensive explorations in CRISPR/Cas9 technology, its specificity ought to be further improved to diminish the off-target risks for safety promise. In general, the term “off-target effect” represents two aspects where the gene editing functions at the non-targeted site in the genome of the targeted cells, and the targeted genes are manipulated in nontargeted tissues or cells. The off-target at the intracellular level is from the repeated expression of Cas9/sgRNA, and the unreasonable design of sgRNA. While the latter off-target effect is mainly ascribed to the lack of cell or tissue selectivity, which could be tackled by biological or delivery strategies. Cell-specific promoters have been constructed in the CRISPR/Cas9 plasmid to solve the issue, and similarly the on-off design could be employed on the translation of Cas9 mRNA and the nuclease activity of Cas9 protein. The nanoparticles-mediated targeting delivery of CRISPR/Cas9 is intensively pursued by many nanomedicine researchers, making it another area that would enhance the appeal of the genome editing technology. In conclusion, the CRISPR/Cas9 system and the corresponding vectors are still in infancy and swiftly evolving. Novel biofunctional vectors should be discovered to accommodate the emerging applications, which will pave the way of CRISPR/Cas9 for the desired therapeutic outcomes.

## Figures and Tables

**Table 1:** CRISPR/Cas9-based clinical trials collected from the ClinicalTrial.gov website (accessed on 20 September 2021).

Target Genes	Condition or Disease	Interventions/ Treatment	Delivery Method	Phase	NCT Identifier
PD-1	lymphoma	universal anti-CD19 CAR-T-cells (CB-010)	unknown (ex vivo)	I	NCT04637763
	metastatic NSCLC	PD-1 knockout T-cells	electroporation	I	NCT02793856
	hepatocellular carcinoma	PD-1 knockout T-cells	unknown (ex vivo)	I	NCT04417764
	Epstein-Barr virus associated malignancies	PD-1 knockout EBV-CTLs	electroporation	I/II	NCT03044743
	esophageal cancer	PD-1 knockout T-cells	unknown (ex vivo)	II	NCT03081715
TCR, PD-1	solid tumor	anti-mesothelin CAR-T-cells	electroporation	I	NCT03545815
TCR, B2M	B-cell leukemia B-cell lymphoma	CAR-T-cells targeting CD19	electroporation	I/II	NCT03166878
TCR, MHC I	renal cell carcinoma	universal anti-CD70 CAR-T-cells (CTX130)	electroporation	I	NCT04438083
	T-cell lymphoma	universal anti-CD70 CAR-T-cells (CTX130)	electroporation	I	NCT04502446
TRAC, β2M	multiple myeloma	universal anti-BCMA CAR-T-cells (CTX120)	electroporation	I	NCT04244656
	B-cell malignancy lymphoma	universal anti-CD19 CAR-T-cells (CTX110)	unknown (ex vivo)	I	NCT04035434
TRAC, CD52	lymphoblastic leukemia	CAR-T-cells targeting CD19	unknown (ex vivo)	I	NCT04557436
CD5	relapsed/refractory hematopoietic malignancies	anti-CD5 CAR-T-cells (CT125A)	unknown (ex vivo)	I	NCT04767308
CD7	high risk T-cell malignancies	CD7-specific CAR-T-cells	unknown (ex vivo)	I	NCT03690011
CD19, CD20 or CD22	B-cell leukemia B-cell lymphoma	universal dual specificity CAR-T-cells	electroporation	I/II	NCT03398967
GISH	gastrointestinal neoplasms	tumor infiltrating lymphocytes	unknown (ex vivo)	I	NCT04426669
HPK1	leukemia lymphocytic	CD19-specific CAR-T-cells	lentivirus and electroporation	I	NCT04037566
BCL11A	β-thalassemia	CD34^+^ HSPCs (CTX001)	electroporation	I/II	NCT03655678
	sickle cell disease	CD34^+^ HSPCs (CTX001)	electroporation	I/II	NCT03745287
	β-thalassemia	CD34^+^ HSPCs (ET-01)	electroporation	I	NCT04925206
β-globin	sickle cell disease	CD34^+^ HSPCs (GPH101)	unknown (ex vivo)	I/II	NCT04819841
HbF	sickle cell disease	HPSCs	unknown (ex vivo)	I/II	NCT04774536
TGF-β receptor II	advanced biliary tract cancer	CAR-EGFR T-cells	unknown (ex vivo)	I	NCT04976218
CCR5	HIV	modified CD34^+^ HSPCs	unknown (ex vivo)	I	NCT03164135
CEP290	retinal disease	photoreceptor cells	adeno-associated virus-5 (in vivo)	I/II	NCT03872479
E6, E7	human papillomavirus-related malignant neoplasm	cervical epithelium	local gel administration (in vivo)	I	NCT03057912

Note: NSCLC: Non-small cell lung cancer; PD-1: Programmed death-1 receptor; EBV: Epstein-Barr virus; CTL: Cytotoxic T-lymphocyte; CAR: Chimeric antigen receptor; TCR: T-cell receptor; MHC I: Major histocompatibility complex class 1; HSPCs: Hematopoietic stem and progenitor cells.

## Data Availability

Not applicable.

## References

[1] Ishino Y., Shinagawa H., Makino K., Amemura M., Nakata A. (1987). Nucleotide sequence of the iap gene, responsible for alkaline phosphatase isozyme conversion in Escherichia coli, and identification of the gene product. J. Bacteriol..

[2] Mojica F.J.M., Diez-Villasenor C., Garcia-Martinez J., Soria E. (2005). Intervening sequences of regularly spaced prokaryotic repeats derive from foreign genetic elements. J. Mol. Evol..

[3] Jinek M., Chylinski K., Fonfara I., Hauer M., Doudna J.A., Charpentier E. (2012). A Programmable Dual-RNA-Guided DNA Endonuclease in Adaptive Bacterial Immunity. Science.

[4] Mali P., Yang L., Esvelt K.M., Aach J., Guell M., DiCarlo J.E., Norville J.E., Church G.M. (2013). RNA-Guided Human Genome Engineering via Cas9. Science.

[5] Cong L., Ran F.A., Cox D., Lin S., Barretto R., Habib N., Hsu P.D., Wu X., Jiang W., Marraffini L.A. (2013). Multiplex Genome Engineering Using CRISPR/Cas Systems. Science.

[6] Palermo G., Chen J.S., Ricci C.G., Rivalta I., Jinek M., Batista V.S., Doudna J.A., McCammon J.A. (2018). Key role of the REC lobe during CRISPR-Cas9 activation by ‘sensing’, ‘regulating’, and ‘locking’ the catalytic HNH domain. Q. Rev. Biophys..

[7] Ran F.A., Hsu P.D., Wright J., Agarwala V., Scott D.A., Zhang F. (2013). Genome engineering using the CRISPR-Cas9 system. Nat. Protoc..

[8] Wang J.B., Exline C.M., DeClercq J.J., Llewellyn G.N., Hayward S.B., Li P.W.L., Shivak D.A., Surosky R.T., Gregory P.D., Holmes M.C. (2015). Homology-driven genome editing in hematopoietic stem and progenitor cells using ZFN mRNA and AAV6 donors. Nat. Biotechnol..

[9] Hsu P.D., Scott D.A., Weinstein J.A., Ran F.A., Konermann S., Agarwala V., Li Y.Q., Fine E.J., Wu X.B., Shalem O. (2013). DNA targeting specificity of RNA-guided Cas9 nucleases. Nat. Biotechnol..

[10] Platt R.J., Chen S., Zhou Y., Yim M.J., Swiech L., Kempton H.R., Dahlman J.E., Parnas O., Eisenhaure T.M., Jovanovic M. (2014). CRISPR-Cas9 Knockin Mice for Genome Editing and Cancer Modeling. Cell.

[11] Mali P., Aach J., Stranges P.B., Esvelt K.M., Moosburner M., Kosuri S., Yang L.H., Church G.M. (2013). CAS9 transcriptional activators for target specificity screening and paired nickases for cooperative genome engineering. Nat. Biotechnol..

[12] Komor A.C., Kim Y.B., Packer M.S., Zuris J.A., Liu D.R. (2016). Programmable editing of a target base in genomic DNA without double-stranded DNA cleavage. Nature.

[13] Gaudelli N.M., Komor A.C., Rees H.A., Packer M.S., Badran A.H., Bryson D.I., Liu D.R. (2017). Programmable base editing of A.T to G.C in genomic DNA without DNA cleavage. Nature.

[14] Cheng A.W., Wang H., Yang H., Shi L., Katz Y., Theunissen T.W., Rangarajan S., Shivalila C.S., Dadon D.B., Jaenisch R. (2013). Multiplexed activation of endogenous genes by CRISPR-on, an RNA-guided transcriptional activator system. Cell Res..

[15] Schumann K., Lin S., Boyer E., Simeonov D.R., Subramaniam M., Gate R.E., Haliburton G.E., Yee C.J., Bluestone J.A., Doudna J.A. (2015). Generation of knock-in primary human T cells using Cas9 ribonucleoproteins. Proc. Natl. Acad. Sci. USA.

[16] Kim K., Ryu S.-M., Kim S.-T., Baek G., Kim D., Lim K., Chung E., Kim S., Kim J.-S. (2017). Highly efficient RNA-guided base editing in mouse embryos. Nat. Biotechnol..

[17] Xu C.F., Chen G.J., Luo Y.L., Zhang Y., Zhao G., Lu Z.D., Czarna A., Gu Z., Wang J. (2021). Rational designs of in vivo CRISPR-Cas delivery systems. Adv. Drug Deliv. Rev..

[18] Li L., Hu S., Chen X. (2018). Non-viral delivery systems for CRISPR/Cas9-based genome editing: Challenges and opportunities. Biomaterials.

[19] Wu Y.X., Zhou H., Fan X.Y., Zhang Y., Zhang M., Wang Y.H., Xie Z.F., Bai M.Z., Yin Q., Liang D. (2015). Correction of a genetic disease by CRISPR-Cas9-mediated gene editing in mouse spermatogonial stem cells. Cell Res..

[20] Xu W. (2019). Microinjection and Micromanipulation: A Historical Perspective. Microinjection.

[21] Gu B., Posfai E., Rossant J. (2018). Efficient generation of targeted large insertions by microinjection into two-cell-stage mouse embryos. Nat. Biotechnol..

[22] Bakhtiar A., Chowdhury E.H. (2021). PH-responsive strontium nanoparticles for targeted gene therapy against mammary carcinoma cells. Asian J. Pharm. Sci..

[23] Kleinstiver B.P., Prew M.S., Tsai S.Q., Nguyen N.T., Topkar V.V., Zheng Z., Joung J.K. (2015). Broadening the targeting range of Staphylococcus aureus CRISPR-Cas9 by modifying PAM recognition. Nat. Biotechnol..

[24] Villiger L., Grisch-Chan H.M., Lindsay H., Ringnalda F., Pogliano C.B., Allegri G., Fingerhut R., Haberle J., Matos J., Robinson M.D. (2018). Treatment of a metabolic liver disease by in vivo genome base editing in adult mice. Nat. Med..

[25] Nelson C.E., Wu Y.Y., Gemberling M.P., Oliver M.L., Waller M.A., Bohning J.D., Robinson-Hamm J.N., Bulaklak K., Rivera R.M.C., Collier J.H. (2019). Long-term evaluation of AAV-CRISPR genome editing for Duchenne muscular dystrophy. Nat. Med..

[26] Kruzik A., Fetahagic D., Hartlieb B., Dorn S., Koppensteiner H., Horling F.M., Scheiflinger F., Reipert B.M., de la Rosa M. (2019). Prevalence of Anti-Adeno-Associated Virus Immune Responses in International Cohorts of Healthy Donors. Mol. Ther.-Methods Clin. Dev..

[27] Merienne N., Vachey G., de Longprez L., Meunier C., Zimmer V., Perriard G., Canales M., Mathias A., Herrgott L., Beltraminelli T. (2017). The Self-Inactivating KamiCas9 System for the Editing of CNS Disease Genes. Cell Rep..

[28] Lombardo A., Cesana D., Genovese P., Di Stefano B., Provasi E., Colombo D.F., Neri M., Magnani Z., Cantore A., Lo Riso P. (2011). Site-specific integration and tailoring of cassette design for sustainable gene transfer. Nat. Methods.

[29] Sanchez-Hernandez S., Gutierrez-Guerrero A., Martin-Guerra R., Cortijo-Gutierrez M., Tristan-Manzano M., Rodriguez-Perales S., Sanchez L., Garcia-Perez J.L., Chato-Astrain J., Fernandez-Valades R. (2018). The IS2 Element Improves Transcription Efficiency of Integration-Deficient Lentiviral Vector Episomes. Mol. Nucleic Acids.

[30] Xu L., Park K.H., Zhao L., Xu J., El Refaey M., Gao Y., Zhu H., Ma J., Han R. (2016). CRISPR-mediated Genome Editing Restores Dystrophin Expression and Function in mdx Mice. Mol. Ther..

[31] Koo T., Yoon A.R., Cho H.Y., Bae S., Yun C.O., Kim J.S. (2017). Selective disruption of an oncogenic mutant allele by CRISPR/Cas9 induces efficient tumor regression. Nucleic Acids Res..

[32] Wang D., Zhang F., Gao G.P. (2020). CRISPR-Based Therapeutic Genome Editing: Strategies and In Vivo Delivery by AAV Vectors. Cell.

[33] Wilbie D., Walther J., Mastrobattista E. (2019). Delivery Aspects of CRISPR/Cas for in Vivo Genome Editing. Acc. Chem. Res..

[34] Branden L.J., Mohamed A.J., Smith C.I.E. (1999). A peptide nucleic acid-nuclear localization signal fusion that mediates nuclear transport of DNA. Nat. Biotechnol..

[35] Chen F., Ding X., Feng Y., Seebeck T., Jiang Y., Davis G.D. (2017). Targeted activation of diverse CRISPR-Cas systems for mammalian genome editing via proximal CRISPR targeting. Nat. Commun..

[36] Zhang L., Wang P., Feng Q., Wang N., Chen Z., Huang Y., Zheng W., Jiang X. (2017). Lipid nanoparticle-mediated efficient delivery of CRISPR/Cas9 for tumor therapy. NPG Asia Mater..

[37] Bai J., Duan J.L., Liu R., Du Y.F., Luo Q., Cui Y.N., Su Z.B., Xu J.R., Xie Y., Lu W.L. (2020). Engineered targeting tLyp-1 exosomes as gene therapy vectors for efficient delivery of siRNA into lung cancer cells. Asian J. Pharm. Sci..

[38] Kim S.M., Yang Y., Oh S.J., Hong Y., Seo M., Jang M. (2017). Cancer-derived exosomes as a delivery platform of CRISPR/Cas9 confer cancer cell tropism-dependent targeting. J. Control Release.

[39] Lin Y., Wu J., Gu W., Huang Y., Tong Z., Huang L., Tan J. (2018). Exosome-Liposome Hybrid Nanoparticles Deliver CRISPR/Cas9 System in MSCs. Adv. Sci..

[40] Li L., Song L., Liu X., Yang X., Li X., He T., Wang N., Yang S., Yu C., Yin T. (2017). Artificial Virus Delivers CRISPR-Cas9 System for Genome Editing of Cells in Mice. ACS Nano.

[41] Wang P., Zhang L., Zheng W., Cong L., Guo Z., Xie Y., Wang L., Tang R., Feng Q., Hamada Y. (2018). Thermo-triggered Release of CRISPR-Cas9 System by Lipid-Encapsulated Gold Nanoparticles for Tumor Therapy. Angew. Chem. Int. Ed. Engl..

[42] Liu B.Y., He X.Y., Xu C., Xu L., Ai S.L., Cheng S.X., Zhuo R.X. (2018). A Dual-Targeting Delivery System for Effective Genome Editing and In Situ Detecting Related Protein Expression in Edited Cells. Biomacromolecules.

[43] Luo Y.L., Xu C.F., Li H.J., Cao Z.T., Liu J., Wang J.L., Du X.J., Yang X.Z., Gu Z., Wang J. (2018). Macrophage-Specific in Vivo Gene Editing Using Cationic Lipid-Assisted Polymeric Nanoparticles. ACS Nano.

[44] Shen J., Lu Z., Wang J., Hao Q., Ji W., Wu Y., Peng H., Zhao R., Yang J., Li Y. (2021). Traceable Nano-Biohybrid Complexes by One-Step Synthesis as CRISPR-Chem Vectors for Neurodegenerative Diseases Synergistic Treatment. Adv. Mater..

[45] Vaidyanathan S., Azizian K.T., Haque A.K.M.A., Henderson J.M., Hendel A., Shore S., Antony J.S., Hogrefe R.I., Kormann M.S.D., Porteus M.H. (2018). Uridine Depletion and Chemical Modification Increase Cas9 mRNA Activity and Reduce Immunogenicity without HPLC Purification. Mol. Ther.-Nucleic Acids.

[46] Anderson B.R., Muramatsu H., Jha B.K., Silverman R.H., Weissman D., Kariko K. (2011). Nucleoside modifications in RNA limit activation of 2’-5’-oligoadenylate synthetase and increase resistance to cleavage by RNase L.. Nucleic Acids Res..

[47] Finn J.D., Smith A.R., Patel M.C., Shaw L., Youniss M.R., van Heteren J., Dirstine T., Ciullo C., Lescarbeau R., Seitzer J. (2018). A Single Administration of CRISPR/Cas9 Lipid Nanoparticles Achieves Robust and Persistent In Vivo Genome Editing. Cell Rep..

[48] Cheng Q., Wei T., Farbiak L., Johnson L.T., Dilliard S.A., Siegwart D.J. (2020). Selective organ targeting (SORT) nanoparticles for tissue-specific mRNA delivery and CRISPR-Cas gene editing. Nat. Nanotechnol..

[49] Farbiak L., Cheng Q., Wei T., Alvarez-Benedicto E., Johnson L.T., Lee S., Siegwart D.J. (2021). All-In-One Dendrimer-Based Lipid Nanoparticles Enable Precise HDR-Mediated Gene Editing In Vivo. Adv. Mater..

[50] Usman W.M., Pham T.C., Kwok Y.Y., Vu L.T., Ma V., Peng B., Chan Y.S., Wei L., Chin S.M., Azad A. (2018). Efficient RNA drug delivery using red blood cell extracellular vesicles. Nat. Commun..

[51] Li Z., Zhou X., Wei M., Gao X., Zhao L., Shi R., Sun W., Duan Y., Yang G., Yuan L. (2019). In Vitro and In Vivo RNA Inhibition by CD9-HuR Functionalized Exosomes Encapsulated with miRNA or CRISPR/dCas9. Nano Lett..

[52] Abbasi S., Uchida S., Toh K., Tockary T.A., Dirisala A., Hayashi K., Fukushima S., Kataoka K. (2021). Co-encapsulation of Cas9 mRNA and guide RNA in polyplex micelles enables genome editing in mouse brain. J. Control. Release.

[53] Javanmardi S., Tamaddon A.M., Aghamaali M.R., Ghahramani L., Abolmaali S.S. (2020). Redox-sensitive, PEG-shielded carboxymethyl PEI nanogels silencing MicroRNA-21, sensitizes resistant ovarian cancer cells to cisplatin. Asian J. Pharm. Sci..

[54] Liu J., Chang J., Jiang Y., Meng X., Sun T., Mao L., Xu Q., Wang M. (2019). Fast and Efficient CRISPR/Cas9 Genome Editing In Vivo Enabled by Bioreducible Lipid and Messenger RNA Nanoparticles. Adv. Mater..

[55] Chen G., Abdeen A.A., Wang Y., Shahi P.K., Robertson S., Xie R., Suzuki M., Pattnaik B.R., Saha K., Gong S. (2019). A biodegradable nanocapsule delivers a Cas9 ribonucleoprotein complex for in vivo genome editing. Nat. Nanotechnol..

[56] Liu C., Wan T., Wang H., Zhang S., Ping Y., Cheng Y. (2019). A boronic acid-rich dendrimer with robust and unprecedented efficiency for cytosolic protein delivery and CRISPR-Cas9 gene editing. Sci. Adv..

[57] Cho E.Y., Ryu J.Y., Lee H.A.R., Hong S.H., Park H.S., Hong K.S., Park S.G., Kim H.P., Yoon T.J. (2019). Lecithin nano-liposomal particle as a CRISPR/Cas9 complex delivery system for treating type 2 diabetes. J. Nanobiotechnol..

[58] Sun W., Ji W., Hall J.M., Hu Q., Wang C., Beisel C.L., Gu Z. (2015). Self-assembled DNA nanoclews for the efficient delivery of CRISPR-Cas9 for genome editing. Angew. Chem. Int. Ed..

[59] Lee K., Conboy M., Park H.M., Jiang F.G., Kim H.J., Dewitt M.A., Mackley V.A., Chang K., Rao A., Skinner C. (2017). Nanoparticle delivery of Cas9 ribonucleoprotein and donor DNA in vivo induces homology-directed DNA repair. Nat. Biomed. Eng..

[60] Lee B., Lee K., Panda S., Gonzales-Rojas R., Chong A., Bugay V., Park H.M., Brenner R., Murthy N., Lee H.Y. (2018). Nanoparticle delivery of CRISPR into the brain rescues a mouse model of fragile X syndrome from exaggerated repetitive behaviours. Nat. Biomed. Eng..

[61] Yin J., Wang Q., Hou S., Bao L., Yao W., Gao X. (2018). Potent Protein Delivery into Mammalian Cells via a Supercharged Polypeptide. J. Am. Chem. Soc..

[62] Gustafsson O., Radler J., Roudi S., Lehto T., Hallbrink M., Lehto T., Gupta D., Andaloussi S.E., Nordin J.Z. (2021). Efficient Peptide-Mediated In Vitro Delivery of Cas9 RNP. Pharmaceutics.

[63] Kang Y.K., Lee J., Im S.H., Lee J.H., Jeong J., Kim D.K., Yang S.Y., Jung K., Kim S.G., Chung H.J. (2021). Cas9 conjugate complex delivering donor DNA for efficient gene editing by homology-directed repair. J. Ind. Eng. Chem..

[64] Rouet R., Thuma B.A., Roy M.D., Lintner N.G., Rubitski D.M., Finley J.E., Wisniewska H.M., Mendonsa R., Hirsh A., de Onate L. (2018). Receptor-Mediated Delivery of CRISPR-Cas9 Endonuclease for Cell-Type-Specific Gene Editing. J. Am. Chem. Soc..

[65] Shen J., Chen J., Ma J., Fan L., Zhang X., Yue T., Yan Y., Zhang Y. (2020). Enhanced lysosome escape mediated by 1,2-dicarboxylic-cyclohexene anhydride-modified poly-l-lysine dendrimer as a gene delivery system. Asian J. Pharm. Sci..

[66] Alsaiari S.K., Patil S., Alyami M., Alamoudi K.O., Aleisa F.A., Merzaban J.S., Li M., Khashab N.M. (2018). Endosomal Escape and Delivery of CRISPR/Cas9 Genome Editing Machinery Enabled by Nanoscale Zeolitic Imidazolate Framework. J. Am. Chem. Soc..

[67] He Q., Chen J., Yan J., Cai S., Xiong H., Liu Y., Peng D., Mo M., Liu Z. (2020). Tumor microenvironment responsive drug delivery systems. Asian J. Pharm. Sci..

[68] Guo J., Wan T., Li B., Pan Q., Xin H., Qiu Y., Ping Y. (2021). Rational Design of Poly(disulfide)s as a Universal Platform for Delivery of CRISPR-Cas9 Machineries toward Therapeutic Genome Editing. ACS Cent. Sci..

[69] Mout R., Ray M., Tonga G.Y., Lee Y.-W., Tay T., Sasaki K., Rotello V.M. (2017). Direct Cytosolic Delivery of CRISPR/Cas9-Ribonucleoprotein for Efficient Gene Editing. ACS Nano.

[70] Kim S.M., Shin S.C., Kim E.E., Kim S.H., Park K., Oh S.J., Jang M. (2018). Simple In Vivo Gene Editing via Direct Self-Assembly of Cas9 Ribonucleoprotein Complexes for Cancer Treatment. ACS Nano.

